# The Impact of Sleep on Neurocognition and Functioning in Schizophrenia—Is It Time to Wake-Up?

**DOI:** 10.20900/jpbs.20220001

**Published:** 2022-01-25

**Authors:** David Kimhy, Luz Ospina, Katie Beck-Felts, Ahmad Fakhoury, Anna E. Mullins, Andrew W. Varga

**Affiliations:** 1 Department of Psychiatry, Icahn School of Medicine at Mount Sinai, New York, NY 10029, USA; 2 New York The Mental Illness Research, Education and Clinical Centers (NY MIRECC), The James J. Peters VA Medical Center, Bronx, NY 10468, USA; 3 Division of Pulmonary, Critical Care and Sleep Medicine, Mount Sinai Integrative Sleep Center, Icahn School of Medicine at Mount Sinai, New York, NY 10029, USA

**Keywords:** sleep, neurocognition, schizophrenia, slow wave sleep, spindles, functioning, psychosis

## Abstract

People with schizophrenia (SZ) display substantial neurocognitive deficits that have been implicated as major contributors to poor daily functioning and disability. Previous reports have identified a number of predictors of poor neurocognition in SZ including demographics, symptoms, and treatment adherence, as well as body mass index, aerobic fitness, and exercise activity. However, the putative impact of sleep has received relatively limited consideration, despite sleep disturbances, which are pervasive in this population, resulting in symptoms that are strikingly similar to the neurocognitive deficits commonly observed in SZ. Here we argue for the consideration of the impact of sleep on neurocognition in people with SZ and propose recommendations for future research to elucidate the links between sleep parameters, neurocognition and daily functioning.

## INTRODUCTION

People with schizophrenia (SZ) display substantial neurocognitive deficits across multiple domains [1,2]. These deficits have been identified as major predictors of poor functioning and disability [2-5], thus representing a serious public health concern and an important target for interventions [6,7]. Studies examining predictors of neurocognitive functioning in SZ have centered on a number of variables including demographics (e.g., age, education, parental education, income, socioeconomic status) [8-11], symptoms (e.g., depression, negative symptoms) [10,12,13], and treatment adherence [12], as well as body mass index (BMI), aerobic fitness, and exercise activity [14-17]. However, as sleep disturbance (SD) symptoms are strikingly similar to the cognitive deficits observed in SZ [18], here we argue for the consideration of the impact of SD on neurocognition and functioning in this population.

## EVIDENCE FOR THE IMPACT OF SLEEP ON NEUROCOGNITION

Extensive preclinical and clinical research literatures converge in highlighting the critical role SD plays in degrading memory and other neurocognitive abilities, pointing to deterioration in multiple cognitive domains [19-22]. Attention and working memory appear to be particularly susceptible to the impact of poor sleep, as evident by a large meta-analysis (70 studies, *N* = 1458) indicating moderate-to-large effect sizes [23]. Previous reports have implicated a number of sleep-related processes as relevant to neurocognitive sequalae. Slow Wave Sleep (SWS), often referred to as “deep sleep”, consists of Stage 3 of non-rapid eye movement (NREM) sleep and is characterized by 0.5–4 Hz high amplitude oscillatory EEG activity [24]. SWS has been associated with both declarative and spatial navigational memory [25-27], with augmentation using transcranial current oscillating at a frequency mimicking SWS during sleep resulting in enhanced declarative memory retention [28]. Similarly, spindle activity has also been implicated in neurocognitive functioning. Spindles are generated by the interplay of the thalamic reticular nucleus, thalamic relay nuclei, and cortex during NREM sleep [29-31] and are characterized by waxing/waning bursts of 10–16 Hz activity. Findings suggests spindles facilitate synaptic plasticity and memory consolidation [24,30,32-36], specifically declarative memory [37], the integration of new information into existing knowledge [38], as well as directed remembering and forgetting [39]. Altogether, these findings point to SWS and spindle activity as promising targets for investigating the neural activity underlying the links between SD and neurocognitive functioning.

## SLEEP IN PEOPLE WITH SCHIZOPHRENIA–CHARACTERISTICS AND IMPACT ON NEUROCOGNITION

Germane to SZ, reports indicate SD are highly prevalent in this population, with nearly 4 out of 5 individuals with SZ endorsing sleep problems [40]. These findings appear to be unrelated to onset and/or chronicity of psychosis, with substantial SD have been documented among individuals at clinical high risk for psychosis [41,42]. Likewise, nearly half of early psychosis patients reported experiences of insomnia and nearly 80% were diagnosed with at least one sleep disorder [43]. A burgeoning research literature supports the link between sleep and neurocognition in SZ [18,44-51], although the findings are not universal, potentially due to diversity of sleep parameters and cognitive domains investigated, as well as a lack of replication [52]. Specifically, results suggest significant associations between SWS and impaired neurocognitive performance in medication-naïve [53], medicated [54-56], and unmedicated individuals with SZ [57,58], with multiple domains being negatively impacted including processing speed and inhibition [49], attention [53,58], procedural learning [56,59], visuospatial memory [55,60], and declarative memory [55,60]. Conversely, extended SWS was found to be correlated with quicker problem solving [61] and enhanced attention [62]. Likewise, reduction of sleep spindle activity has been linked with impaired neurocognition in individuals with SZ [50,63-65] including impaired memory consolidation [64], inattention [53,66], and verbal cognition [67]. Augmenting spindles in isolation did not improve sleep-dependent memory processes in SZ [68], speaking to the need for their precise temporal coordination with slow oscillations during SWS [69].

## IMPLICATIONS FOR FUTURE RESEARCH

Despite their high prevalence and clinical significance, at present there are limited data on the impact of SD on neurocognition in SZ and there are no data quantifying their influence on daily functioning. The extant studies have been limited by small samples, reliance of cross-sectional designs, as well as evaluations of a narrow range of sleep parameters and neurocognitive domains. Additionally, the common use of retrospective self-reports, which are susceptible to various cognitive biases in individuals with SZ [70], is also problematic. Most notably, there have been no experimental studies examining the impact of SD on neurocognition and functioning in people with SZ, limiting the ability to determine whether SD are a cause or an effect of clinical phenomena. Altogether, the available data indicates that despite their chronic and ubiquitous nature, SD remain poorly understood and modeled in SZ, their impact is rarely considered in clinical trials targeting neurocognitive deficits, and they remain largely unaddressed by clinicians.

Addressing this critical gap in knowledge would require employment of a multi-pronged approach. Specifically, here we propose recommendations for investigators to employ in future studies examining the links between sleep and poor neurocognition in SZ:

1. A greater emphasis should be placed on employment of experimental studies over correlational and/or cross-sectional designs. Such a strategy could clarify and confirm the causal links between neurobiological, physiological, and subjective characteristics of sleep and neurocognition. Recent reports have recommended the use of controlled sleep deprivation as an experimental model of SZ [71]. Likewise, pharmacological approaches (e.g., Ketamine) have been used to model sleep in SZ [72-74].
2. Consistent with the National Institute of Mental Health (NIMH)’s Research Domain Criteria (RDoC) framework, studies would benefit from concurrent assessments of multiple domains using complementary methodologies (e.g., polysomnography, electroencephalogram (EEG), behavioral, subjective, ambulatory assessments, as well as imaging).
3. Studies should aim to identify sleep parameters impacting poor neurocognition, as well as explore whether any specific neurocognitive domains may be more susceptible for deterioration due to poor sleep.
4. The scope of outcomes should be expanded beyond traditional neurocognitive test batteries to include measures of daily functioning, social cognition, and quality of life.
5. Studies should aim to ascertain the impact of sleep on biomarkers associated with poor neurocognition in SZ (e.g., neurotrophins, inflammation markers).
6. Finally, it is imperative future studies examine putatively relevant biological variables (e.g., sex, age, BMI, menstrual cycle a menopause) to ascertain their potential impact on the link between sleep and neurocognition in SZ.

Our group is currently undertaking such a study aiming to address these very questions (PI: Kimhy; “*Neurocognition After Perturbed Sleep (NAPS)*”; ClinicalTrials.gov Identifier: NCT05032963).
